# Risk Stratification and Validation of Eleven Autophagy-Related lncRNAs for Esophageal Squamous Cell Carcinoma

**DOI:** 10.3389/fgene.2022.894990

**Published:** 2022-06-27

**Authors:** Xu Zhao, Yulun Wang, Fanbiao Meng, Zhuang Liu, Bo Xu

**Affiliations:** ^1^ Department of Biochemistry and Molecular Biology, Key Laboratory of Breast Cancer Prevention and Therapy, Ministry of Education, Tianjin Medical University Cancer Institute and Hospital, National Clinical Research Center for Cancer, Key Laboratory of Cancer Prevention and Therapy, Tianjin’s Clinical Research Center for Cancer, Tianjin Medical University, Tianjin, China; ^2^ Center for Intelligent Oncology, Chongqing Key Laboratory of Intelligent Oncology for Breast Cancer, Chongqing University Cancer Hospital, Chongqing University School of Medicine, Chongqing, China

**Keywords:** esophageal squamous cell carcinoma, autophagy, prognosis, risk model, long noncoding RNA

## Abstract

Esophageal squamous cell carcinoma (ESCC), the most prevalent subtype of esophageal cancer, ranks sixth in cancer-related mortality, making it one of the deadliest cancers worldwide. The identification of potential risk factors for ESCC might help in implementing precision therapies. Autophagy-related lncRNAs are a group of non-coding RNAs that perform critical functions in the tumor immune microenvironment and therapeutic response. Therefore, we aimed to establish a risk model composed of autophagy-related lncRNAs that can serve as a potential biomarker for ESCC risk stratification. Using the RNA expression profile from 179 patients in the GSE53622 and GSE53624 datasets, we found 11 lncRNAs (AC004690.2, AC092159.3, AC093627.4, AL078604.2, BDNF-AS, HAND2-AS1, LINC00410, LINC00588, PSMD6-AS2, ZEB1-AS1, and LINC02586) that were co-expressed with autophagy genes and were independent prognostic factors in multivariate Cox regression analysis. The risk model was constructed using these autophagy-related lncRNAs, and the area under the receiver operating characteristic curve (AUC) of the risk model was 0.728. To confirm that the model is reliable, the data of 174 patients from The Cancer Genome Atlas (TCGA) esophageal cancer dataset were analyzed as the testing set. A nomogram for ESCC prognosis was developed using the risk model and clinic-pathological characteristics. Immune function annotation and tumor mutational burden of the two risk groups were analyzed and the high-risk group displayed higher sensitivity in chemotherapy and immunotherapy. Expression of differentially expressed lncRNAs were further validated in human normal esophageal cells and esophageal cancer cells. The constructed lncRNA risk model provides a useful tool for stratifying risk and predicting the prognosis of patients with ESCC, and might provide novel targets for ESCC treatment.

## Introduction

Esophageal cancer (EC) has an increasingly notable cancer burden, accounting for approximately 16% (Ferlay et al., 2015) cancer-related mortality worldwide (Smyth et al., 2017). According to GLOBOCAN estimates, over 604,100 new cases of EC and 544,076 EC-related deaths occurred in 2020 (Thrift, 2021). The two main histological subtypes of EC are esophageal squamous cell carcinoma (ESCC) and esophageal adenocarcinoma (EAC). In all EC cases, the proportion of ESCC was >90%. Although its incidence has declined over the past decades, the survival ratios for EC are among the lowest for cancers, mainly because of the late stage at diagnosis and high aggressiveness of the disease. The fatality rate of ESCC is even higher than that of EAC, with a 5-year overall survival rate of <15%. Hence, there is an urgent need to search for effective screening methods and risk stratification to improve patient prognoses.

In the transcriptome, less than 1–2% of RNAs encode proteins and undergo the translation process. Thus, most RNAs are non-coding (Beermann et al., 2016); among these, RNAs with more than 200 nucleotides are called long non-coding RNAs (lncRNAs) (Djebali et al., 2012; Dykes and Emanueli, 2017). Compared with protein-coding messenger RNAs, whose functions are extensively studied, the specific function of most lncRNAs remains unknown (Johnson et al., 2005). lncRNAs are reported to be crucial in many biological processes, including epigenetic modification, cell cycle regulation, and differentiation (Beermann et al., 2016). Although the mechanism by which lncRNAs regulate physiological activities is unclear, their significance, especially in tumor growth and metastasis, has been reported (Quinn and Chang, 2016; Li et al., 2018; Chi et al., 2019).

Autophagy has become a popular research topic since Yoshinori Ohsumi was awarded the Nobel Prize in Physiology or Medicine for his contribution in elucidating its mechanism in 2016. Thus, stimulation or inhibition of autophagy in cancer cells has also become a promising therapeutic strategy (Levy et al., 2017). Considering the complexity of various biological processes, the role of autophagy in tumors can be both positive and negative (Russo and Russo, 2018). Autophagy can be metaphorized as a double-edged sword (Pietrocola et al., 2016). In the specific cellular microenvironments of certain tumors (Galluzzi et al., 2015), autophagy can either promote or inhibit cancer development. Despite these paradoxical approaches, there are no reports on autophagy-related lncRNAs as prognostic biomarkers for patients with ESCC.

In this study, using transcriptional and clinical data from two databases, TCGA database and the Gene Expression Omnibus (GEO) datasets, GSE53622 and GSE53624, we first constructed an autophagy-associated lncRNA model and validated its prognostic value. Immune function, tumor mutation burden (TMB), and therapeutic response of the two risk groups were further explored. To determine the expression level of autophagy-related lncRNAs in cultured human cells, the selected lncRNAs were analyzed using quantitative real-time polymerase chain reaction (qRT-PCR).

## Methods

### Esophageal Squamous Cell Carcinoma Clinical and Transcriptional Datasets

The clinical data and lncRNA expression profiles of 179 ESCC patients in the GSE53622 and GSE53624 datasets were obtained from the GEO database and re-annotated using the GPL18109 platform. Data from 174 patients with EC were obtained from TCGA (https://cancergenome.nih.gov/) and used for independent external validation.

### Screening of Autophagy-Related Genes and Long Non-Coding RNAs Screening

After annotation and categorization using GENCOED (https://www.gencodegenes.org), all extracted mRNA and lncRNA expression profiles were compared against the HaDb website, an online database dedicated to collecting ARGs and proteins (http://autophagy.lu/clustering/index.html). After autophagy-related mRNAs were selected, correlations between autophagy-associated mRNAs and their co-expressed lncRNAs were analyzed using the Pearson method, and the screening criteria were |R^2^ | > 0.3 and *p* < 0.001. Autophagy-related lncRNAs were defined based on these criteria. Cytoscape was used to visualize the correlation network of autophagy genes and their associated lncRNAs. All mRNA sequencing data were standardized using the limma package (version 3.22.7).

### Construction of a Prognostic Autophagy Long Non-Coding RNAs Model

Using univariate Cox regression analysis, 43 lncRNAs were identified from all selected autophagy-related lncRNAs. Further multivariate regression analysis revealed the statistically significant prognostic autophagy-associated lncRNAs, which were used in constructing the model. Based on the coefficients of these lncRNAs, the patient’s risk value was calculated formula as follows:

$$\mathrm{Risk score}=\beta_{1}X_{1}+\beta_{2}X_{2}+\cdots +\beta_{n}X_{n}$$

The β value represents the regression coefficient of each lncRNA, and the X value represents its transcriptional expression. To increase the accuracy of this risk assessment formula, lncRNA expression levels were weighted using regression coefficients for the linear combination of allocating risk scores. The β value was obtained by the logarithmic transformation of the HR value. After evaluating the risk values of all patients, they were classified into high or low groups based on the median risk value.

### Assessment and External Validation of the Prognostic Model

Kaplan-Meier (KM) survival curves of the high-and low-risk groups were plotted to compare the overall survival of the two groups. Based on the clinical data of the GSE53624 and GSE53622 training sets, the receiver operator characteristic curve (ROC) of clinical features such as age, sex, grade, and other characteristics were plotted, and the predictive ability of each feature was evaluated by the area under the curve (AUC). SPSS software was used for statistical analysis and statistical significance was set at *p* < 0.05. Using the same standards and methods, TCGA dataset was obtained as the testing set to further confirm the stability and reliability of the model.

Nomogram was generated including risk scores and other clinical characteristics with R package of “survival”, “regplot” and “rms”. Calibration curves of 1-year survival, 3-year survival and 5-year survival were delineated.

### Functional Analysis

Through Kyoto Encyclopedia of Genes and Genomes (KEGG) pathway analysis, we found that different signaling pathways were enriched in different groups. KEGG pathway analysis was implemented based on a gene-set enrichment analysis (GSEA) software, which was downloaded from the website, https://www.gsea-msigdb.org/gsea/index.jsp. A false discovery set of 1000 repeats, *p*-value < 0.05, and q-FDR < 0.25 were considered valid. The gene ontology (GO) database was used for gene and gene product classifications.

### Immune Function Heatmap and Tumor Mutational Burden Analysis

Differential analysis of immune-associated genes in the high or low risk groups of patients were performed and visualized using the R package “ssGSEA”. Simple nucleotide variation (SNV) profile of the TCGAdataset was downloaded to analyze the tumor mutational burden (TMB) in high or low risk group. Survival curves of different TMB and risk subgroups were analyzed.

### Therapeutic Response Analysis

The “pRRophetic” package was used to estimate the therapeutic sensitivity of patients in high or low risk group based on half maximal inhibitory concentration (IC50) of anticancer drugs in the Cancer Genome Project (CGP) database. Filtering criteria was *p* < 0.05.

### Cell Culture

Human normal esophageal cells (HEEpiC) and esophageal cancer cell lines KYSE30 and KYSE150 were cultured according to the manufacturer’s instructions (Procell, Wuhan, China) in RPMI-1640 (Solarbio, Beijing, China) supplemented with 10% fetal bovine serum, at 37°C with 5% carbon dioxide.

### Differential Validation Using Quantitative Real-Time Polymerase Chain Reaction

TRIZOL reagent (Invitrogen, Grand Island, NY, United States) was used to extract total RNA from cultured cells plated in a 6-well plate. The RNA concentration and quality were determined using a NanoDrop spectrophotometer (Thermo Fisher Scientific, MA, United States). The extracted total RNA was reverse-transcribed into cDNA. qRT-PCR was performed according to the manufacturer’s instructions (Takara-Bio), using the following primer sequences: HAND2-AS1, 5′-CGGTCCCTAGCAACAAGGTT-3′ (F) and 5′-CTTTCTGCGCTTACACCTGG-3′ (R); ZEB1-AS1, 5′-TTGGGCGATTTTGAAGTGCG-3′ (F) and 5′-GTGGAGAGGACTGGTTTCGG-3′ (R). The relative lncRNA expression was calculated using the formula 2^−ΔΔCt^. The experiment was performed three times.

### Statistical Analysis

All calculations and visualization of bioinformatic data were performed using R language software (version x64 4.1.3, survival library), including generation of Kaplan-Meier survival curves, univariate and multivariate regression analyses, calculation of risk values, plotting risk heat maps and multi-catalog ROC curves, and evaluation of AUC values. GSEA (version 4.0.3) was used to visualize functional enrichment distinctions between the high-and low-risk groups of patients with ESCC. The statistical significance of each test was set at a separation value of *p* < 0.05.

## Results

### Screening of Prognostic Long Non-Coding RNAs

Among the 179 patients with ESCC, 146 were male and 33 were female. The median patient age was 59 years. The histological distribution of these 179 patients was as follows: 10 patients were stage I, 77 were stage II, and 92 were stage III. The median overall survival was 2.81 years. Based on the combined clinical outcomes of these 179 patients, 43 prognostic-related lncRNAs were obtained after screening using univariate Cox regression (Table 1). Of these, 11 lncRNAs were identified as independent prognostic lncRNAs using multivariate Cox regression, including AC004690.2, AC092159.3, AC093627.4, AL078604.2, BDNF-AS, HAND2-AS1, LINC00410, LINC00588, PSMD6-AS2, ZEB1-AS1, and LINC02586 (Table 2).

**TABLE 1:** Significant prognostic autophagy lncRNAs in ESCC patients.

LncRNA	KM	B	SE	HR	HR.95L	HR.95H	*p*-value
AC004690.2	0.003646	−0.21326	0.102314	0.807949	0.661141	0.987357	0.037131
AC009135.1	0.005497	0.165645	0.071065	1.180154	1.026711	1.356528	0.019758
AC010745.1	0.044803	0.153274	0.075702	1.165644	1.004913	1.352083	0.042898
AC011365.1	0.018151	0.276166	0.132672	1.318066	1.016264	1.709495	0.037382
AC011365.2	0.023922	0.302114	0.136334	1.352716	1.035522	1.767072	0.026692
AC012494.1	0.012603	0.762773	0.257307	2.144214	1.294935	3.550492	0.003032
AC017074.2	0.039703	−0.46976	0.159679	0.625153	0.457159	0.854881	0.003262
AC026412.3	0.007657	0.53665	0.250314	1.710268	1.04712	2.793393	0.03204
AC079349.1	0.049192	−0.33239	0.110128	0.717207	0.577968	0.88999	0.002542
AC079943.1	0.016194	0.566801	0.230095	1.762619	1.122798	2.76704	0.013765
AC090061.1	0.023284	0.457723	0.160335	1.580471	1.154275	2.164032	0.004306
AC092159.3	0.012035	0.276189	0.121963	1.318097	1.037845	1.674027	0.023541
AC093627.4	0.012721	−0.39403	0.140017	0.674332	0.512496	0.887272	0.00489
AC138123.2	0.007043	0.255316	0.121419	1.290869	1.017491	1.637699	0.035485
AC245297.3	0.015701	−0.2887	0.117788	0.749235	0.59478	0.9438	0.014245
AC254629.1	0.013056	0.93071	0.409419	2.53631	1.136853	5.658486	0.023011
AL078604.2	0.014692	−0.3034	0.1246	0.738301	0.578327	0.942526	0.014891
AL135960.1	0.011216	−0.37666	0.17255	0.686148	0.489264	0.962261	0.029042
AL137026.2	0.02769	0.287181	0.086905	1.332666	1.123952	1.580137	0.000951
AL139130.1	0.011762	−0.23579	0.089401	0.789948	0.66298	0.941232	0.008354
AL512631.2	0.035362	0.216095	0.101459	1.241221	1.01739	1.514296	0.033181
AL590068.1	0.025632	0.253603	0.109691	1.28866	1.039367	1.597747	0.020779
BDNF−AS	0.029138	0.909921	0.413291	2.484127	1.105044	5.584292	0.02769
C5orf66	0.027392	−0.87709	0.424911	0.415992	0.180884	0.956689	0.039002
CRNDE	0.007695	0.672485	0.314376	1.9591	1.057936	3.627887	0.032427
HAND2-AS1	0.017144	−0.74111	0.354538	0.476582	0.237878	0.954819	0.036585
LINC00395	0.027561	0.358055	0.12449	1.430545	1.120818	1.82586	0.004025
LINC00410	0.021815	−0.24107	0.118117	0.785785	0.623394	0.990479	0.041255
LINC00588	0.045615	−0.52531	0.233579	0.591374	0.374144	0.934729	0.024516
LINC01003	0.026687	−0.37946	0.190036	0.684232	0.47146	0.993029	0.04585
LINC02305	0.021849	−0.45951	0.167896	0.63159	0.454488	0.877705	0.006202
LINC02586	0.027443	−0.30574	0.155866	0.736575	0.54268	0.999746	0.04981
PSMD6-AS2	0.012729	−0.52696	0.20008	0.590394	0.398872	0.873879	0.008444
RARA-AS1	0.011405	−0.26451	0.128956	0.76758	0.596151	0.988306	0.040249
SNHG3	0.00561	0.585328	0.237023	1.795579	1.128365	2.857322	0.013531
SNHG5	0.043276	−0.70253	0.320349	0.495329	0.26437	0.928058	0.028306
TRBV11-2	0.036456	0.171816	0.086587	1.187459	1.002111	1.407088	0.04722
ZEB1-AS1	0.015706	0.619751	0.276488	1.858465	1.080955	3.195223	0.024993
ZIM2-AS1	0.005903	−0.42966	0.204767	0.650731	0.435616	0.972075	0.03588
ATG7	0.041523	0.488018	0.245711	1.629083	1.006453	2.636896	0.047017
DAPK2	0.049092	0.361962	0.143164	1.436145	1.084767	1.901341	0.011462
NAF1	0.027078	0.274767	0.098221	1.316224	1.085736	1.595642	0.005151
RGS19	0.003052	0.468488	0.165387	1.597577	1.155271	2.209225	0.004616

**TABLE 2:** The 11 autophagy-related lncRNAs significant by multivariate Cox analysis.

LncRNA	Coefficients	HR
AC004690.2	−0.30132	0.739838
AC092159.3	0.405486	1.500032
AC093627.4	−0.60338	0.546961
AL078604.2	−0.38601	0.679762
BDNF-AS	1.393802	4.030144
HAND2-AS1	−1.24773	0.287157
LINC00410	0.279909	1.32301
LINC00588	−0.52222	0.593205
PSMD6-AS2	−0.83716	0.43294
ZEB1-AS1	0.906655	2.476026
LINC02586	−0.26822	0.764741

### Co-Expression Diagram of Autophagy-Related Long Non-Coding RNAs

**FIGURE 1:**
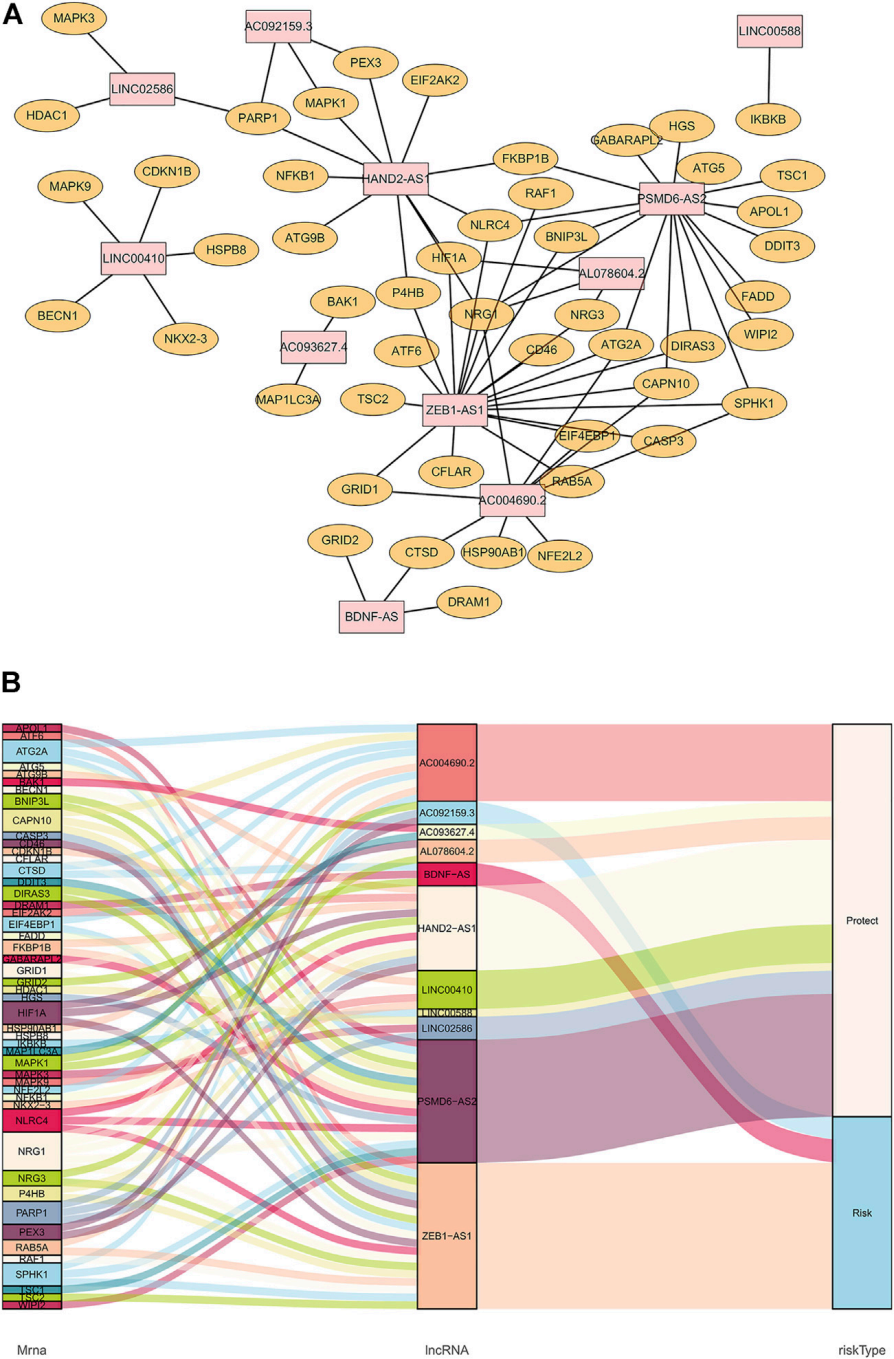
Co-expression of autophagy-related genes and lncRNAs. **(A)**: Orange nodes are autophagy-related genes, and pink nodes are their related prognostic lncRNAs. The network was generated by Cytoscape 3.7.2. **(B)**: Sankey diagram of autophagy-related genes, lncRNAs, and their risk types in patients’ prognosis.

The co-expression diagram of the 11 prognostic-related lncRNAs and ARGs is shown in Figure 1A. To describe the crosstalk between lncRNAs and ARGs as well as its role in patient survival outcomes, we constructed a Sankey diagram (Figure 1B). The results showed that patients with ESCC showing high AC092159.3, BDNF-AS, or ZEB1-AS1 expression levels were at a higher risk of poor prognosis. In contrast, patients with high AC004690.2, AC093627.4, AL078604.2, HAND2-AS1, LINC00410, LINC00588, LINC02586, or PSMD6-AS2 expression had a lower risk of longer overall survival.

**FIGURE 2:**
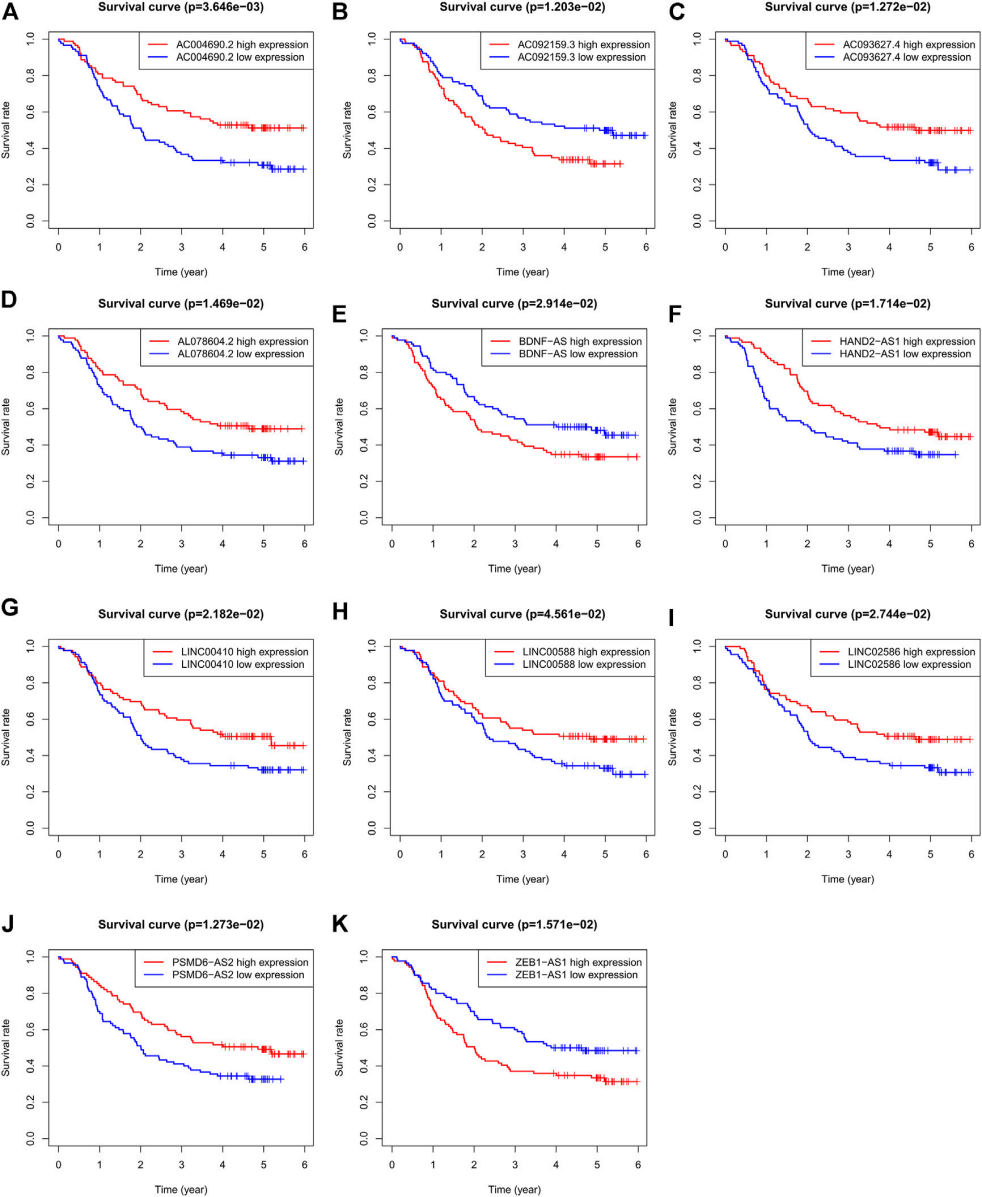
KM analysis of the 11 autophagy-related lncRNAs in GSE63625 ESCC patients. **(A)**: OS curves of ESCC patients based on AC004690.2 expression. **(B)**: OS curves of ESCC patients based on AC092159.3 expression. **(C)**: OS curves of ESCC patients based on AC093627.4 expression. **(D)**: OS curves of ESCC patients based on AL078604.2 expression. **(E)**: OS curves of ESCC patients based on BDNF-AS expression. **(F)**: OS curves of ESCC patients based on HAND2-AS1 expression. **(G)**: OS curves of ESCC patients based on LINC00410 expression. **(H)**: OS curves of ESCC patients based on LINC00588 expression. **(I)**: OS curves of ESCC patients based on LINC02586 expression. **(J)**: OS curves of ESCC patients based on PSMD6-AS2 expression. **(K)**: OS curves of ESCC patients based on ZEB1-AS1 expression.

To validate the ability of the 11 candidate lncRNAs to predict patient prognosis, KM analysis was performed using the survival data of patients with ESCC, as shown in Figure 2. Based on the Sankey analysis, patients with high AC092159.3, BDNF-AS, or ZEB1-AS1 expression showed significantly shorter survival with a lower median overall survival, whereas patients with high AC004690.2, AC093627.4, AL078604.2, HAND2-AS1, LINC00410, LINC00588, LINC02586, or PSMD6-AS2 expression had a lower risk and longer overall survival.

### Development of a Prognostic Autophagy Long Non-Coding RNAs Signature in Esophageal Squamous Cell Carcinoma

**FIGURE 3:**
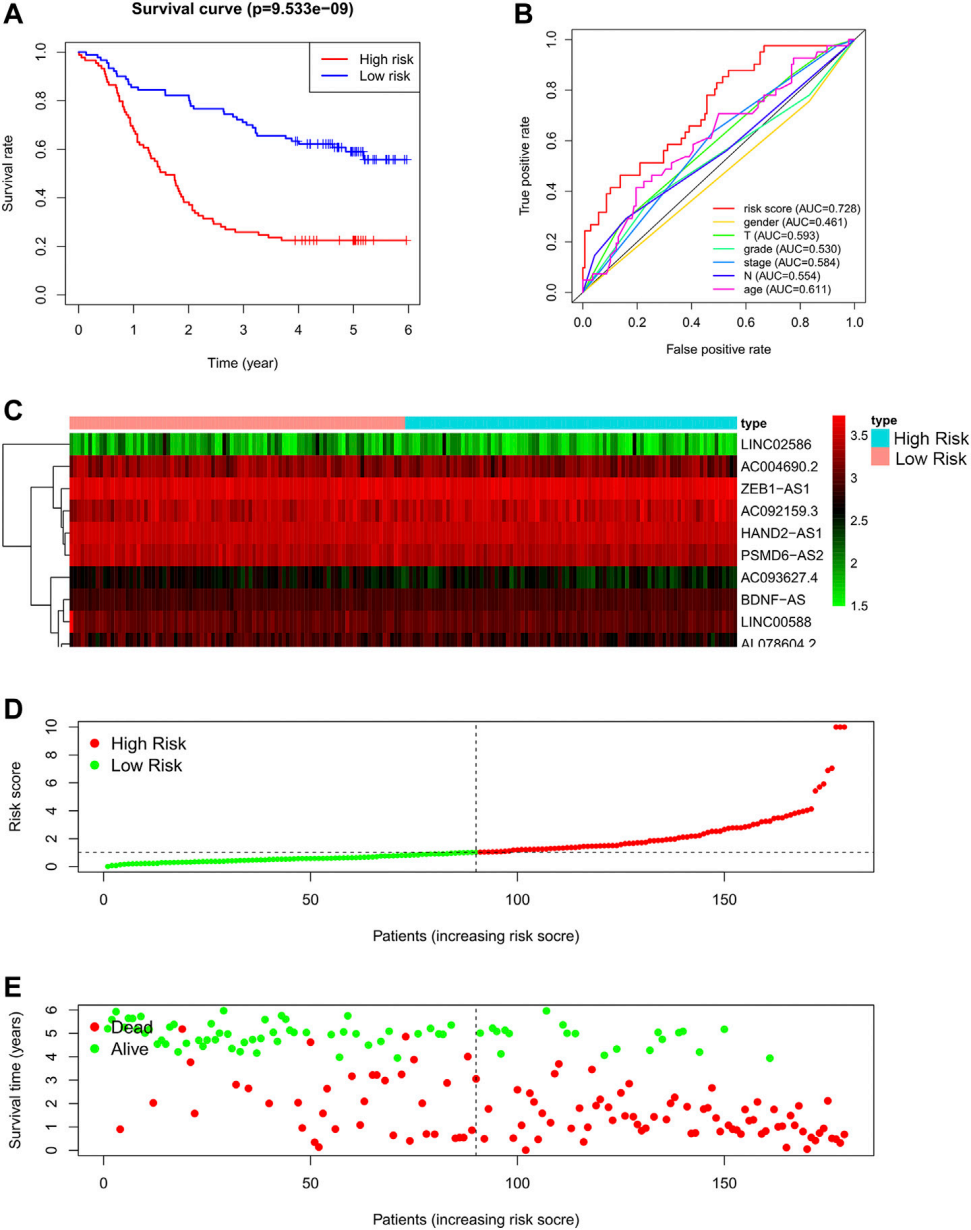
Autophagy related lncRNA risk model in the GSE63625 training set. **(A)**: Survival curve of ESCC patients based on the risk value, **(B)**: ROC of the risk model compared with other clinical characteristics, **(C)**: Expression heatmap of the 11 lncRNAs in patients, **(D)**: Patients distribution based on the risk value, **(E)**: Survival status of all ESCC patients.

Based on the derivation equation, we comprehensively determined the risk value of every patient by multiplying the expression levels of the 11 lncRNAs with their correlation coefficients. Depending on the median value of the risk score, patients were categorized into high-or low-risk groups, and the prognosis of both groups was compared using Kaplan-Meier analysis (Figure 3A). We found that patients in the high-risk group had significantly worse outcomes, both in terms of survival and duration of survival, than those in the low-risk group. The 5-year survival rate of the high-risk group was approximately 20%, compared with 60% in the low-risk group (*p* < 0.0001).

To evaluate the performance of the risk model, we also drew a multi-index ROC curve (Figure 3B), with an AUC value of 0.728, which was higher than that of other clinical characteristics, indicating that the constructed risk model has the best value for predicting the outcome of patients with ESCC. Furthermore, the risk curve, in addition to the heat map of all patients, is shown in Figures 3C–E. As shown in Figures 3B–D, as the risk score of patients increases, the survival rate of patients decreases.

### External Validation

**FIGURE 4:**
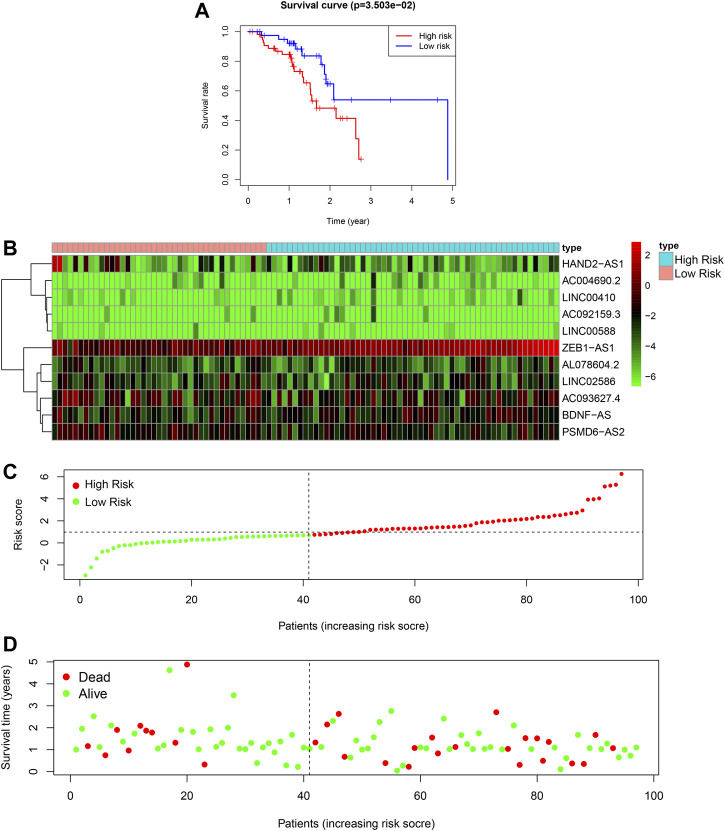
Autophagy related lncRNA risk model in the TCGA testing set. **(A)**: Survival curve of ESCC patients based on the risk value, **(B)**: Expression heatmap of the 11 lncRNAs in patients, **(C)**: Patients distribution based on the risk value, **(D)**: Survival status of all ESCC patients.

To validate the model, we used data from patients in TCGA database as an external testing set. The risk analysis of the testing set, and KM survival curve are shown in Figures 4A–C. In the KM survival curve, the two groups were distinguished, and the *p*-value was less than 0.05. Our constructed risk model for autophagy and prognostic lncRNAs was thus validated as a significant prognostic indicator.

### Independent Prognostic Analysis and Nomogram

**FIGURE 5:**
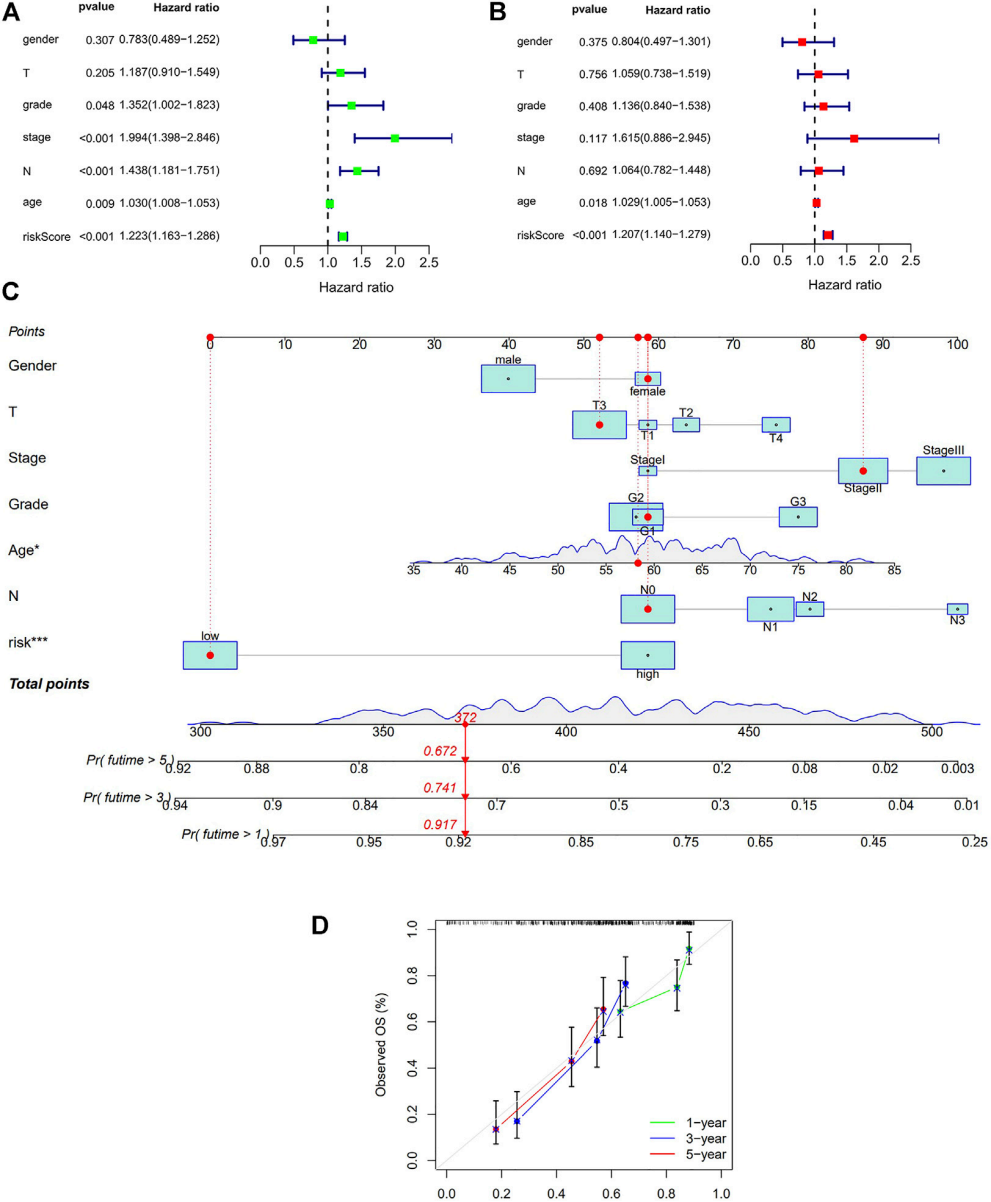
Cox regression analysis and Nomogram in the training set. **(A)**: Univariate Cox regression of risk model and other clinical factors in the training set **(B)**: Multivariate Cox regression of risk model and other clinical factors in the training set **(C)**: Nomogram for both clinic-pathological factors and prognostic autohphagy-related lncRNAs. The value of each variable corresponds to a score on the point scale axis. A total point can be calculated by adding all scores according to each patient’s situation and projected to the risk scale. **(D)**: Calibration curves for the nomogram. The x-axis represents the nomogram predicted OS and y-axis represents observed OS in reality. Perfect prediction would correspond to the 45-degree gray line.

Finally, we conducted univariate and multivariate prognostic analyses based on the risk values in both the training and testing sets. The results showed that in the univariate regression test (Figures 5A,B), the *p*-value was <0.001 and the HR value was 1.224, whereas in the multivariate regression, the *p*-value was <0.001 and the HR value was 1.191. Briefly, independent prognostic analysis, whether univariate or multivariate, confirmed that our established risk model can be an independent risk indicator to precisely evaluate the outcome of patients with ESCC. In order to obtain a more accurate evaluation tool for predicting each patient’s prognosis, we combined the autophagy-related lncRNA risk model with other clinical characteristics and built a nomogram as shown in Figures 5C–D.

### Gene Set Enrichment Analysis

**FIGURE 6:**
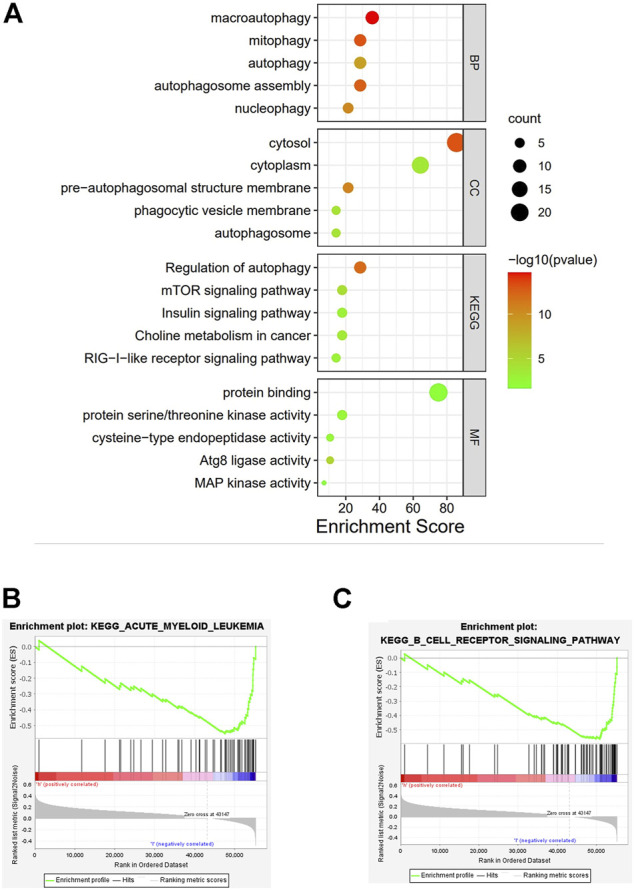
**(A)**: GO analysis of the risk model. **(B,C)**: KEGG pathway enrichment of the risk model.

Using GO annotation and KEGG pathway enrichment, the lncRNAs were found to be enriched in 19 pathways with 50 annotated terms; the top5 GO annotations are shown in Figure 6A. GO enrichment analysis showed that the selected lncRNAs were mainly involved in biological functions associated with various autophagy processes in cells, as well as autophagy-related signaling pathways. The other related pathways included mTOR signaling pathways, insulin signaling pathways, and choline metabolic signaling pathways (Figure 6A). To further investigate the underlying molecular interaction network of the screened features in esophageal squamous cell carcinoma, the gene sets were analyzed using GSEA. The filter criteria were *p*-value < 0.05 and q-FDR value <0.25 (Figures 6B,C). Different enrichments resulted in significantly different risk sets. As shown in Figure 6B, the B cell receptor signaling pathway and Acute myeloid leukemia pathway were significantly enriched in the low-risk set, which was connected with immune regulation, suggesting that activation of the B cell receptor signaling pathway can regulate immune function in the low-risk set, thus predicting a better prognosis and longer survival time. Unfortunately, in the high-risk set, we did not obtain distinct enrichment results, suggesting that the group with a low-risk score was associated with activated immune function. These data provide potential for further research on the personalized treatment of ESCC.

### Immune Function Heatmap and Tumor Mutational Burden

**FIGURE 7:**
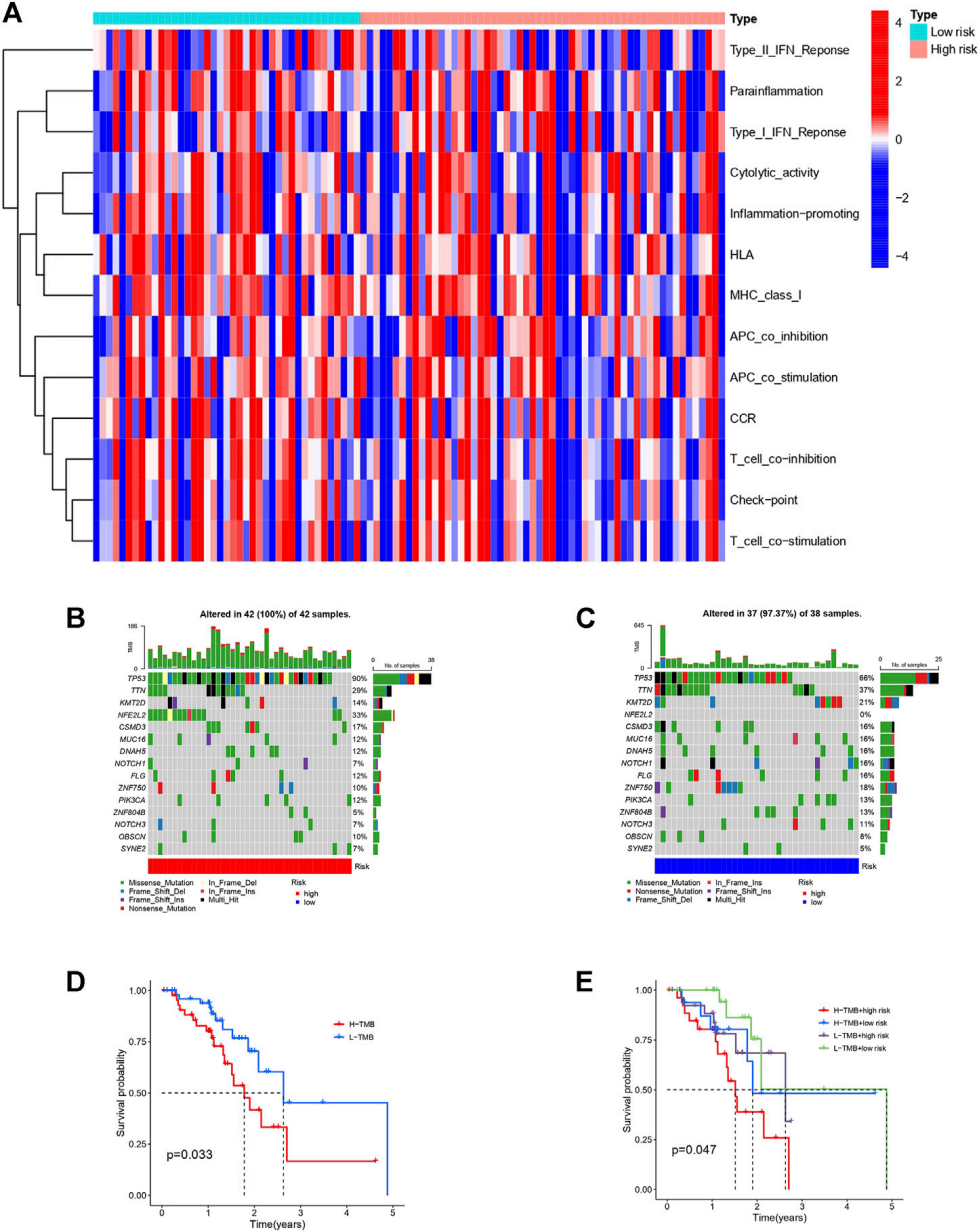
Immune function heatmap and TMB in high and low risk groups of patients with ESCC. **(A)**: Heatmap of immune functions of the two risk groups. **(B)**: Waterfall plot of TMB in high-risk group. **(C)**: Waterfall plot of TMB in low-risk group. **(D)**: Survival curves of patients with ESCC divided into high or low-TMB groups. **(E)**: Survival curves of patients with different TMB based on high or low risk classification.

It has been reported that autophagy plays certain role in mediating innate and adaptive immune responses. In the KEGG and GO analyses we noticed enrichments in cellular autophagy and immunity. Therefore, we compared the difference of immune functions of the high and low risk groups and the heatmap is shown in Figure 7A. Several attempts have been made to identify predictive biomarkers for immunotherapy response. One of the most intriguing and divisive is TMB. Hence, we explored the TMB of the high and low risk group as shown in Figures 7B–C. Generally, the frequency of mutation is higher in the high-risk group and we also found the majority mutation in the high-risk group was associated with mismatch-repair deficiency. Patients with high TMB had shorter OS and unfavorable prognosis. Survival analyses combining TMB and risk model of patients with ESCC is shown in Figures 7D–E.

### Chemotherapy and Immunotherapy Sensitivity

**FIGURE 8:**
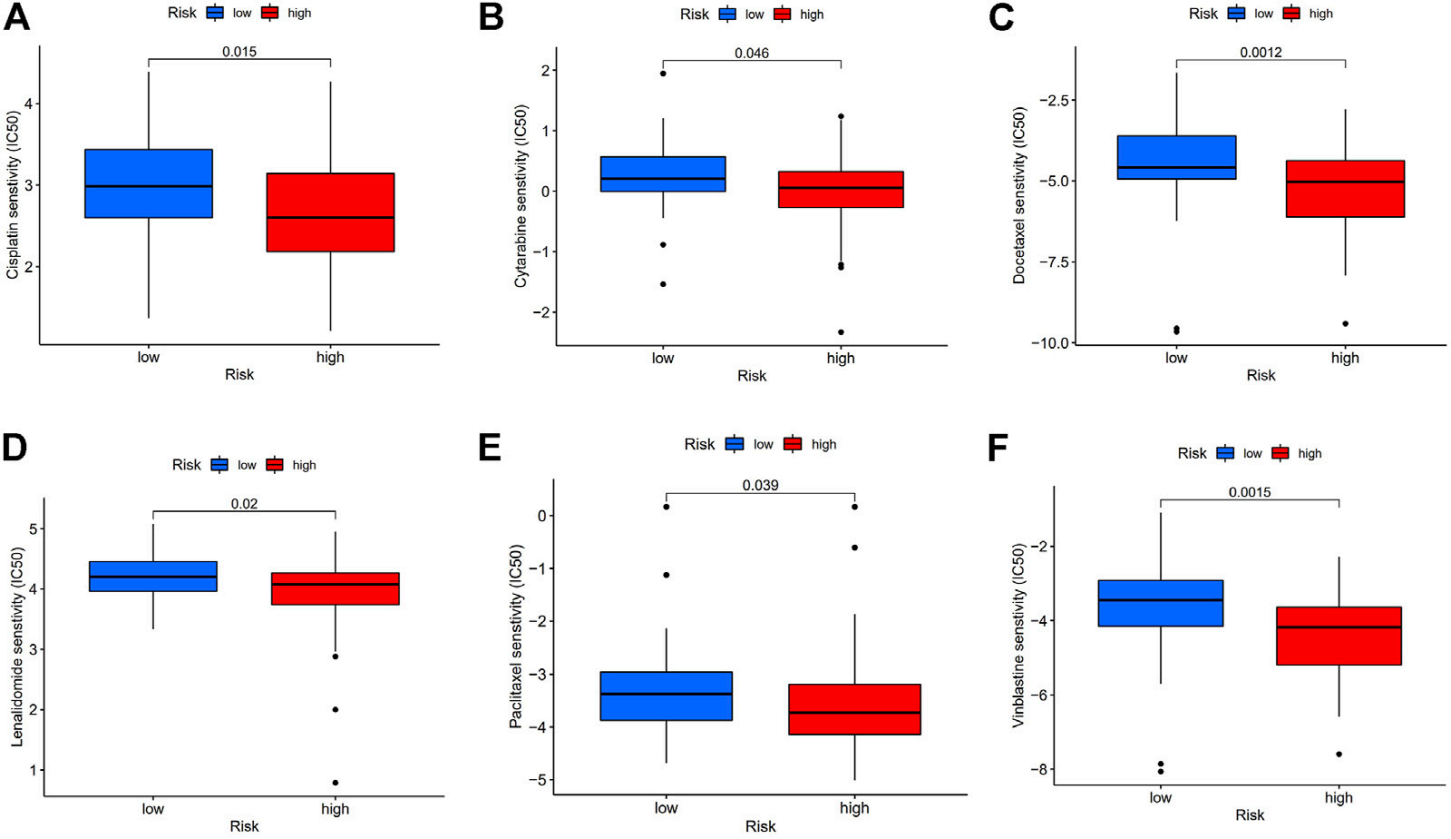
Chemotherapy and immunotherapy response. **(A–F)**: Sensitivity to Cisplatin, Cytarabine, Docetaxel, Paclitaxel, Vinblastine, and Lenalidomide in high or low-risk group shown in box plot.

Therapeutic response analysis showed patients with ESCC in the high-risk group showed lower IC50 in five commonly used chemotherapy drugs for cancer treatment (Cisplatin, Cytarabine, Docetaxel, Paclitaxel, and Vinblastine) (Figure 8), indicating that the high-risk patients are more sensitive to chemotherapy. We also found a lower IC50 of Lenalidomide in the high-risk group, suggesting that these patients are more likely to benefit from Lenalidomide immunotherapy.

### Expression of Signature Long Non-Coding RNAs in Esophageal Squamous Cell Carcinoma Cell Lines by Quantitative Real-Time Polymerase Chain Reaction

**FIGURE 9:**
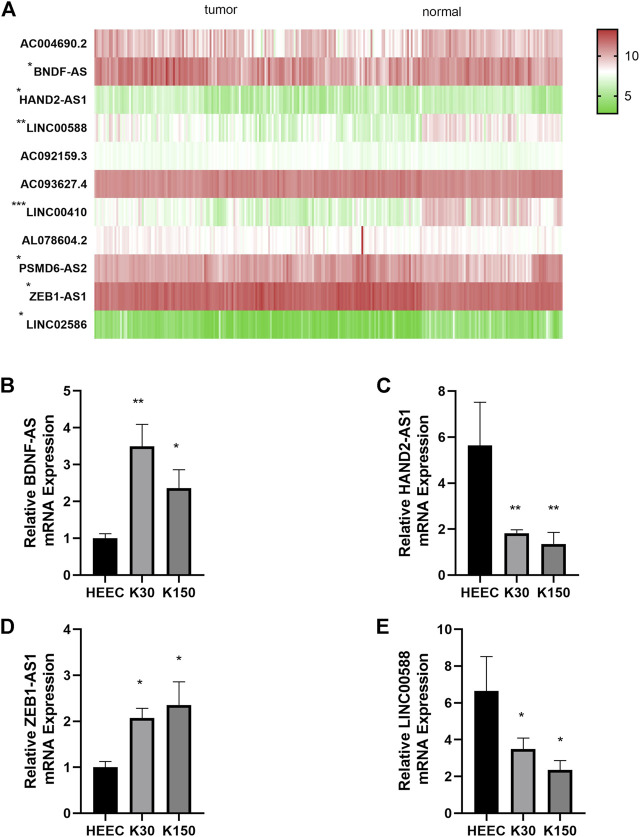
Differential expression of autophagy-related lncRNAs. **(A)**: Differential expression of seven autophagy-related lncRNAs in cancer tissue vs. normal tissue **(B)**: Relative BDNF-AS1 expression in HEEC, K30, K150 cells. **(C)**: Relative HAND2-AS1 expression in HEEC, K30, K150 cells. **(D)**: Relative ZEB1-AS1 expression in HEEC, K30, K150 cells. **(E)**: Relative LINC00588 expression in HEEC, K30, K150 cells.

Differential expression of the 11 autophagy-realted lncRNAs in cancer versus normal tissue is shown in Figure 9A. 7 (BDNF-AS, HAND2-AS1, LINC00410, LINC00588, PSMD6-AS2, ZEB1-AS1, and LINC02586) of the 11 lncRNAs were differentially expressed in cancer and normal tissues. Here we selected four sequence-available lncRNAs (BDNF-AS, HAND2-AS1, LINC00588, and ZEB1-AS1) of the seven differentially expressed lncRNAs with qRT-PCR. As shown in Figures 9B–E, the expression levels of HAND2-AS1 and LINC00588 were significantly lower in KYSE30 and KYSE150 cells than that in HEEpiC, *p* < 0.05, whereas the expression levels of BNDF-AS and ZEB1-AS1 were higher in KYSE30 and KYSE150 compared to that in HEEpiC cells. The remaining three lncRNAs could not be quantified because of a lack of transcriptome information in NCBI.

## Discussion

In 2018, more than 572,000 patients were newly diagnosed with EC (Bray et al., 2018). Treatment of EC remains a challenge because of its recurrence and unfavorable prognosis. The overall 5-year survival rate of EC can be as low as 20% owing to the advanced stage at diagnosis (mainly stage III or IV) and its high invasiveness (Anderson et al., 2015). Although the regional distribution of the two main pathological subtypes has changed over the past 4 decades, ESCC still accounts for the majority of EC cases (Siegel et al., 2019). Therefore, understanding the epidemiological characteristics and risk factors of EC is crucial for public health and clinical decision making, including risk assessment, disease screening, and prevention. The current research on risk stratification mainly focuses on screening Barrett’s esophagus and gastroesophageal reflux disease (GERD) symptom rating scales (Rubenstein et al., 2013; Dong et al., 2018; Rubenstein et al., 2020). A valid risk stratification tool for ESCC thus remains lacking.

Autophagy is a highly conserved pathway that plays a crucial role in both normal and cancerous cells. Multiple cancers exhibit disrupted autophagy regulation. Autophagy is responsible for alterations in cancer cell metabolic regulation and plays a critical role in promoting immune escape. Targeting the autophagy mechanisms remains a promising strategy for the treatment of an increasing number of cancers. Emerging evidence suggests that lncRNAs may also play a crucial role in tumorigenesis (Liu et al., 2022a; Liu et al., 2022b). Recently, Zhang et al. (2020) reported a co-expression pattern of lncRNA HOTAIR and MTHFR, which regulate the biological behavior of EC cells. According to Hu et al. (2021), lncRNA RP11-465B22.8 can be delivered to macrophages *via* exosomes to induce the M2 phenotype, thus enhancing the migration and invasion of EC cells. Their research indicates that lncRNAs are a novel target and based on their regulation of immunity in EC they can potentially provide new therapeutic strategies. In a study by Wu et al. (2022), 22 autophagy-related lncRNAs were included in the risk assessment of patients with EAC, but none of these lncRNAs were identical to those included our stratification model. This may be caused by differences in histological classification. Prognostic lncRNAs in other cancer types have also been reported in recent years, including breast cancer (Wang J. et al., 2019), colon cancer (Zhou et al., 2020), pancreatic cancer (Deng et al., 2020), and bladder cancer (Lai et al., 2020). Most of these models have been constructed based on public databases, with other datasets used for validation. The AUC value varied from 0.6 to 0.9, and was statistically significant.

In a previous study (Shi et al. (2021), also reported a prognostic model in ESCC consisted of nine autophagy-related lncRNAs. It is noticed that the 11 lncRNA signatures in our model had no overlap with theirs. This may due to different statistical approach and filtering criteria. In their study, three of the nine lncRNAs for model construction were independent prognostic factors. In comparison, all 11 lncRNAs in our model were significant by multivariate Cox analyses and the model was externally validated using other databases in addition to differential expression validation by qRT-PCR. In this study, we used autophagy-associated lncRNAs as prognostic stratification biomarkers to screen for ESCC risk. Validation in an independent database showed that the AUC value (0.647) of our signature was higher than that of other clinical characteristics. Kaplan-Meier analysis showed that the two risk groups based on our risk stratification model showed different prognoses, with a *p* < 0.001. A nomogram for predicting patients’ OS was built with the risk model and clinic-pathological features. Further TMB and therapeutic response analyses displayed significant distinctions between the two risk groups. Among the 11 lncRNAs screened for our risk model, seven (BDNF-AS, HAND2-AS1, LINC00410, LINC00588, PSMD6-AS2, ZEB1-AS1, and LINC02586) were found to be differentially expressed in adjacent normal tissues and cancer tissues. Of these, four (BDNF-AS, HAND2-AS1, LINC00588, and ZEB1-AS1) lncRNAs were further quantified in cultured human ESCC cells and normal epithelial cells; the results obtained were consistent with our data analysis. Unfortunately, the remaining three lncRNAs could not be quantified because of a lack of transcriptome information in NCBI.

Among the autophagy-related lncRNAs in our risk model, differential expression of the following three lncRNAs, BDNF-AS1, HAND2-AS1, and ZEB1-AS1, in normal and cancer tissues is commonly found in several cancer types with critical roles in cancer progression. In colon cancer, low expression of BDNF-AS1 can upregulate glycogen synthase kinase-3+, thereby inhibiting the proliferation, invasion, and metastatic ability of colon cancer cells (Zhi and Lian, 2019). In esophageal cancer cell lines, BDNF-AS1 can co-regulate mir-214 and thus regulating the growth and invasion of esophageal cancer cells. Further, BDNF-AS1 has diverse effects on other cancer types, mostly inhibiting epithelial-mesenchymal transition (EMT) and tumor formation in tumor cells (Cao et al., 2010). The lncRNA HAND2-AS1 inhibits tumorigenesis and is expressed in various tumor tissues at lower levels than in adjacent normal tissues (Wang Y. et al., 2019). Abnormal HAND2-AS1 expression is associated with tumor progression and prognosis. Reduced HAND2-AS1 is reported to inhibit cancer growth and correlates with clinical features such as lymph node involvement, histological differentiation (Yang et al., 2017), tumor size, and staging. ZEB1-AS1 is localized on chromosome 10p11.22 with two exons and one intron in between (Gao et al., 2019), and is derived from the ZEB1 promoter (Jiao et al., 2020). Its subcellular localization is in the nucleus. ZEB1-AS1was identified early for its prominent role in promoting cancer cell proliferation (Chen and Shen, 2020). Further, ZEB1-AS1 expression is found to be significantly higher in hepatocellular tumor tissues than in normal tumor-adjacent tissues, and is abnormally elevated in metastatic HCC tissues. High ZEB1-AS1 expression has also been detected in various hepatocellular carcinoma cell lines.

The limitation of our research is that the enrolled patients’ data were mainly obtained from public databases. A larger number of patients with follow-up data are needed to further validate the performance of the model. Moreover, the AUC of our model could potentially be improved by integrating multi-omics data if available. However, the biological functions and underlying mechanisms of most lncRNAs remain elusive.

To our knowledge, this study presents the first autophagy-associated lncRNA signature for risk assessment in ESCC. This autophagy-related lncRNA model provides an effective means for predicting the prognosis of patients with ESCC; moreover, some of these lncRNAs might also serve as novel targets for ESCC treatment.

## Data Availability

The original contributions presented in the study are included in the article/supplementary material, further inquiries can be directed to the corresponding author.

## Nomenclature

### Abbreviations

ESCC: esophageal squama cell carcinoma
LncRNA: long non-coding RNAs
AUC: the area under the receiver operating
ARG: autophagy related gene
TMB: tumor mutation burden
CGP: the cancer genome project
EC: esophageal cancer
EAC: esophageal adenocarcinoma
GEO: gene expression omnibus
TCGA: cancer genome atlas
HADb: human autophagy database
The KM curves: the kaplan meier survival curves
ROC: the receiver operator characteristic curve
KEGG: kyoto encyclopedia of genes and genomes
GO: gene ontology
GSEA: gene set enrichment analysis
HEEpiC: human normal esophageal cells
K30: KYSE30
K150: KYSE150
